# 4-Methyl­phenyl 4-bromo­benzoate

**DOI:** 10.1107/S1600536811040426

**Published:** 2011-10-08

**Authors:** Rodolfo Moreno-Fuquen, Javier Ellena, Carlos A. De Simone

**Affiliations:** a Departamento de Química – Facultad de Ciencias, Universidad del Valle, Apartado 25360, Santiago de Cali, Colombia; b Instituto de Física, IFSC, Universidade de São Paulo, São Carlos, Brazil

## Abstract

In the title compound, C_14_H_11_BrO_2_, an ester formed from the reaction of 4-methyl­phenol with 4-bromo­benzoyl­chloride, the dihedral angle between the benzene rings is 54.43 (7)°, indicating a twist in the mol­ecule. In the crystal, weak C—H⋯O inter­actions link the mol­ecules into supra­molecular layers in the *bc* plane, and these are connected along the *a* axis by Br⋯Br contacts [3.6328 (5) Å].

## Related literature

For industrial applications of ester systems, see: Gowda *et al.* (2007*a* ▶); Brüning *et al.* (2009 ▶). For related structures, see: Gowda *et al.* (2007*b* ▶, 2008 ▶). For hydrogen bonding, see: Nardelli (1995 ▶). For halogen inter­actions, see: Ritter (2009 ▶).
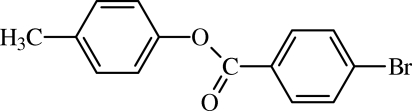


## Experimental

### 

#### Crystal data

C_14_H_11_BrO_2_
*M* *_r_* = 291.13
Monoclinic, 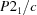

*a* = 15.0219 (9) Å
*b* = 11.3585 (8) Å
*c* = 7.5077 (4) Å
β = 99.730 (4)°
*V* = 1262.58 (14) Å^3^
*Z* = 4
Mo *K*α radiation
μ = 3.24 mm^−1^
*T* = 293 K
0.47 × 0.18 × 0.10 mm

#### Data collection

Bruker–Nonius KappaCCD diffractometer
Absorption correction: multi-scan (*SADABS*; Sheldrick, 1996 ▶) *T*_min_ = 0.472, *T*_max_ = 0.698
9090 measured reflections
2829 independent reflections
1811 reflections with *I* > 2σ(*I*)
*R*_int_ = 0.052

#### Refinement

*R*[*F* ^2^ > 2σ(*F* ^2^)] = 0.042
*wR*(*F* ^2^) = 0.125
*S* = 1.01
2829 reflections
156 parameters
H-atom parameters constrained
Δρ_max_ = 0.31 e Å^−3^
Δρ_min_ = −0.36 e Å^−3^

### 

Data collection: *COLLECT* (Nonius, 2004 ▶); cell refinement: *SCALEPACK* (Otwinowski & Minor, 1997 ▶); data reduction: *DENZO* (Otwinowski & Minor, 1997 ▶) and *SCALEPACK*; program(s) used to solve structure: *SHELXS97* (Sheldrick, 2008 ▶); program(s) used to refine structure: *SHELXL97* (Sheldrick, 2008 ▶); molecular graphics: *ORTEP-3 for Windows* (Farrugia, 1997 ▶); software used to prepare material for publication: *WinGX* (Farrugia, 1999 ▶) and *PARST* (Nardelli, 1995 ▶).

## Supplementary Material

Crystal structure: contains datablock(s) I, global. DOI: 10.1107/S1600536811040426/tk2795sup1.cif

Structure factors: contains datablock(s) I. DOI: 10.1107/S1600536811040426/tk2795Isup2.hkl

Supplementary material file. DOI: 10.1107/S1600536811040426/tk2795Isup3.cml

Additional supplementary materials:  crystallographic information; 3D view; checkCIF report

## Figures and Tables

**Table 1:** Hydrogen-bond geometry (Å, °)

*D*—H⋯*A*	*D*—H	H⋯*A*	*D*⋯*A*	*D*—H⋯*A*
C3—H3⋯O1^i^	0.93	2.67	3.483 (4)	147
C13—H13⋯O1^ii^	0.93	2.77	3.422 (4)	128

Symmetry codes: (i) 
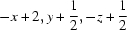
; (ii) 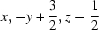.

**Fig. 1.**
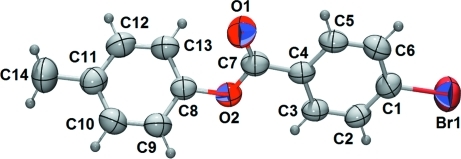
Molecular structure of (I) with displacement ellipsoids drawn at the 50% probability level. H atoms are shown as spheres of arbitrary radius.

## supplementary crystallographic information

### Comment

This work is part of the study of the effect of substituents on the ester system. Similar work, involving ester systems with an emphasis on industrial applications has been published (Gowda *et al.*, 2007*a*; Brüning *et al.*, 2009). The molecular structure of the title compound (I) is similar to that of 4-bromophenyl benzoate (4BPB) (Gowda *et al.*, 2008) and 4-methylphenyl 4-methylbenzoate (4MPB) (Gowda *et al.*, 2007*b*). Compound (I) shows a dihedral angle of 54.43 (7)° between the mean planes of the benzene rings (Fig. 1). This dihedral angle is close to the values presented in the 4BPB and 4MPB molecules [58.43 (17) and 60.17 (7)°, respectively].

The crystal packing is stabilized by C—H···O interactions (Nardelli, 1995); Table 1. These weak interactions link the molecules into supramolecular layers in the *bc* plane. The layers are connected by Br···Br interactions (Ritter, 2009).

### Experimental

A solution containing equimolar quantities (3.4 mmol) of 4-bromobenzoyl chloride and 4-methylphenol in acetonitrile (60 ml) was gradually heated to reflux for 2 h and then allowed to cool. At room temperature, triethylamine was added to get a solid which was poured in cold water. The solid was recrystallized from its dichlorometane solution to yield colourless crystals; *M*.pt. 385 (1) K.

### Refinement

The H-atoms were positioned geometrically [C—H = 0.93–0.96 Å, and with *U*_iso_(H) =1.2–1.5*U*_eq_(C).

### Crystal data

**Table:**

C_14_H_11_BrO_2_	*F*(000) = 584
*M**_r_* = 291.13	*D*_x_ = 1.532 Mg m^−^^3^
Monoclinic, *P*2_1_/*c*	Melting point: 385(1) K
Hall symbol: -P 2ybc	Mo *K*α radiation, λ = 0.71073 Å
*a* = 15.0219 (9) Å	Cell parameters from 9005 reflections
*b* = 11.3585 (8) Å	θ = 2.9–27.5°
*c* = 7.5077 (4) Å	µ = 3.24 mm^−^^1^
β = 99.730 (4)°	*T* = 293 K
*V* = 1262.58 (14) Å^3^	Prism, colourless
*Z* = 4	0.47 × 0.18 × 0.10 mm

### Data collection

**Table:**

Bruker–Nonius KappaCCD diffractometer	2829 independent reflections
Radiation source: fine-focus sealed tube	1811 reflections with *I* > 2σ(*I*)
horizonally mounted graphite crystal	*R*_int_ = 0.052
Detector resolution: 9 pixels mm^-1^	θ_max_ = 27.5°, θ_min_ = 3.7°
CCD scans	*h* = −19→19
Absorption correction: multi-scan (*SADABS*; Sheldrick, 1996)	*k* = −14→10
*T*_min_ = 0.472, *T*_max_ = 0.698	*l* = −9→9
9090 measured reflections	

### Refinement

**Table:**

Refinement on *F*^2^	Secondary atom site location: difference Fourier map
Least-squares matrix: full	Hydrogen site location: inferred from neighbouring sites
*R*[*F*^2^ > 2σ(*F*^2^)] = 0.042	H-atom parameters constrained
*wR*(*F*^2^) = 0.125	*w* = 1/[σ^2^(*F*_o_^2^) + (0.0577*P*)^2^ + 0.3548*P*] where *P* = (*F*_o_^2^ + 2*F*_c_^2^)/3
*S* = 1.01	(Δ/σ)_max_ < 0.001
2829 reflections	Δρ_max_ = 0.31 e Å^−^^3^
156 parameters	Δρ_min_ = −0.36 e Å^−^^3^
0 restraints	Extinction correction: *SHELXL97* (Sheldrick, 2008), Fc^*^=kFc[1+0.001xFc^2^λ^3^/sin(2θ)]^-1/4^
Primary atom site location: structure-invariant direct methods	Extinction coefficient: 0.020 (3)

### Special details

**Table:**

Geometry. All esds (except the esd in the dihedral angle between two l.s. planes) are estimated using the full covariance matrix. The cell esds are taken into account individually in the estimation of esds in distances, angles and torsion angles; correlations between esds in cell parameters are only used when they are defined by crystal symmetry. An approximate (isotropic) treatment of cell esds is used for estimating esds involving l.s. planes.
Refinement. Refinement of F^2^ against ALL reflections. The weighted R-factor wR and goodness of fit S are based on F^2^, conventional R-factors R are based on F, with F set to zero for negative F^2^. The threshold expression of F^2^ > 2sigma(F^2^) is used only for calculating R-factors(gt) etc. and is not relevant to the choice of reflections for refinement. R-factors based on F^2^ are statistically about twice as large as those based on F, and R- factors based on ALL data will be even larger.

### Fractional atomic coordinates and isotropic or equivalent isotropic displacement parameters (Å^2^)

**Table:**

	*x*	*y*	*z*	*U*_iso_*/*U*_eq_	
Br1	0.61020 (2)	0.93326 (4)	0.00395 (6)	0.0970 (2)	
C14	1.4444 (2)	0.8443 (4)	0.4571 (6)	0.1050 (13)	
H14A	1.4685	0.8902	0.5615	0.158*	
H14B	1.4718	0.8687	0.3564	0.158*	
H14C	1.4572	0.7625	0.4817	0.158*	
O2	1.06435 (14)	0.91018 (16)	0.2775 (3)	0.0640 (5)	
O1	1.02822 (15)	0.7422 (2)	0.4071 (3)	0.0864 (7)	
C5	0.8438 (2)	0.7772 (2)	0.2633 (4)	0.0615 (7)	
H5	0.8594	0.7079	0.3270	0.074*	
C1	0.7324 (2)	0.9019 (3)	0.0998 (4)	0.0631 (7)	
C3	0.8867 (2)	0.9613 (2)	0.1444 (4)	0.0590 (6)	
H3	0.9309	1.0156	0.1275	0.071*	
C2	0.7978 (2)	0.9834 (3)	0.0749 (4)	0.0619 (7)	
H2	0.7815	1.0526	0.0116	0.074*	
C8	1.15716 (19)	0.8889 (2)	0.3280 (3)	0.0558 (6)	
C7	1.0054 (2)	0.8281 (3)	0.3177 (4)	0.0617 (7)	
C4	0.91105 (18)	0.8576 (2)	0.2403 (3)	0.0545 (6)	
C6	0.7555 (2)	0.7987 (3)	0.1938 (4)	0.0663 (7)	
H6	0.7111	0.7444	0.2095	0.080*	
C11	1.3432 (2)	0.8623 (3)	0.4124 (4)	0.0723 (8)	
C13	1.1971 (2)	0.7893 (2)	0.2718 (4)	0.0641 (7)	
H13	1.1624	0.7313	0.2056	0.077*	
C9	1.2087 (2)	0.9753 (3)	0.4252 (4)	0.0636 (7)	
H9	1.1814	1.0422	0.4630	0.076*	
C12	1.2891 (2)	0.7777 (3)	0.3156 (4)	0.0710 (8)	
H12	1.3161	0.7103	0.2789	0.085*	
C10	1.3008 (2)	0.9615 (3)	0.4656 (4)	0.0720 (8)	
H10	1.3355	1.0202	0.5302	0.086*	

### Atomic displacement parameters (Å^2^)

**Table:**

	*U*^11^	*U*^22^	*U*^33^	*U*^12^	*U*^13^	*U*^23^
Br1	0.0691 (3)	0.1091 (4)	0.1086 (4)	0.00332 (19)	0.00328 (19)	0.0103 (2)
C14	0.067 (2)	0.107 (3)	0.140 (3)	0.002 (2)	0.014 (2)	0.004 (3)
O2	0.0677 (12)	0.0511 (11)	0.0726 (11)	0.0013 (9)	0.0099 (9)	0.0076 (9)
O1	0.0789 (14)	0.0797 (15)	0.0998 (15)	0.0048 (12)	0.0129 (11)	0.0395 (13)
C5	0.0802 (19)	0.0442 (14)	0.0603 (14)	−0.0036 (13)	0.0130 (13)	0.0073 (11)
C1	0.0668 (16)	0.0635 (17)	0.0598 (15)	0.0025 (14)	0.0127 (12)	−0.0013 (13)
C3	0.0727 (17)	0.0450 (14)	0.0630 (14)	−0.0011 (12)	0.0221 (13)	0.0035 (11)
C2	0.0724 (17)	0.0500 (15)	0.0656 (15)	0.0088 (14)	0.0187 (13)	0.0065 (13)
C8	0.0665 (16)	0.0492 (14)	0.0530 (13)	0.0001 (12)	0.0137 (11)	0.0047 (11)
C7	0.0760 (18)	0.0527 (15)	0.0577 (14)	−0.0008 (14)	0.0145 (12)	0.0066 (13)
C4	0.0695 (16)	0.0446 (14)	0.0507 (13)	0.0012 (12)	0.0141 (11)	0.0017 (10)
C6	0.0751 (18)	0.0569 (17)	0.0677 (15)	−0.0106 (14)	0.0144 (13)	0.0014 (13)
C11	0.0743 (18)	0.066 (2)	0.0790 (18)	0.0034 (15)	0.0196 (15)	0.0049 (15)
C13	0.0816 (19)	0.0510 (15)	0.0596 (14)	0.0004 (14)	0.0118 (13)	−0.0032 (12)
C9	0.0733 (18)	0.0506 (15)	0.0700 (16)	−0.0020 (14)	0.0212 (13)	−0.0066 (13)
C12	0.086 (2)	0.0580 (17)	0.0729 (17)	0.0097 (16)	0.0240 (15)	−0.0012 (14)
C10	0.078 (2)	0.0616 (18)	0.0766 (18)	−0.0107 (15)	0.0145 (15)	−0.0076 (14)

### Geometric parameters (Å, °)

**Table:**

Br1—C1	1.889 (3)	C3—H3	0.9300
C14—C11	1.515 (5)	C2—H2	0.9300
C14—H14A	0.9600	C8—C9	1.380 (4)
C14—H14B	0.9600	C8—C13	1.380 (4)
C14—H14C	0.9600	C7—C4	1.477 (4)
O2—C7	1.355 (3)	C6—H6	0.9300
O2—C8	1.402 (3)	C11—C10	1.386 (5)
O1—C7	1.200 (3)	C11—C12	1.383 (5)
C5—C6	1.363 (4)	C13—C12	1.372 (4)
C5—C4	1.394 (4)	C13—H13	0.9300
C5—H5	0.9300	C9—C10	1.375 (4)
C1—C6	1.382 (4)	C9—H9	0.9300
C1—C2	1.384 (4)	C12—H12	0.9300
C3—C2	1.373 (4)	C10—H10	0.9300
C3—C4	1.397 (4)		
			
C11—C14—H14A	109.5	O1—C7—C4	124.7 (3)
C11—C14—H14B	109.5	O2—C7—C4	112.1 (2)
H14A—C14—H14B	109.5	C5—C4—C3	119.0 (3)
C11—C14—H14C	109.5	C5—C4—C7	118.0 (2)
H14A—C14—H14C	109.5	C3—C4—C7	123.0 (2)
H14B—C14—H14C	109.5	C5—C6—C1	119.4 (3)
C7—O2—C8	118.6 (2)	C5—C6—H6	120.3
C6—C5—C4	120.9 (3)	C1—C6—H6	120.3
C6—C5—H5	119.6	C10—C11—C12	117.3 (3)
C4—C5—H5	119.6	C10—C11—C14	122.6 (3)
C6—C1—C2	120.9 (3)	C12—C11—C14	120.1 (3)
C6—C1—Br1	119.9 (2)	C12—C13—C8	118.5 (3)
C2—C1—Br1	119.2 (2)	C12—C13—H13	120.7
C2—C3—C4	120.2 (3)	C8—C13—H13	120.7
C2—C3—H3	119.9	C10—C9—C8	119.3 (3)
C4—C3—H3	119.9	C10—C9—H9	120.3
C3—C2—C1	119.6 (3)	C8—C9—H9	120.3
C3—C2—H2	120.2	C13—C12—C11	122.6 (3)
C1—C2—H2	120.2	C13—C12—H12	118.7
C9—C8—C13	120.7 (3)	C11—C12—H12	118.7
C9—C8—O2	117.6 (2)	C9—C10—C11	121.6 (3)
C13—C8—O2	121.5 (3)	C9—C10—H10	119.2
O1—C7—O2	123.3 (3)	C11—C10—H10	119.2
			
C4—C3—C2—C1	−0.3 (4)	O2—C7—C4—C3	−2.8 (4)
C6—C1—C2—C3	0.1 (4)	C4—C5—C6—C1	−0.2 (4)
Br1—C1—C2—C3	179.9 (2)	C2—C1—C6—C5	0.2 (4)
C7—O2—C8—C9	−128.0 (3)	Br1—C1—C6—C5	−179.6 (2)
C7—O2—C8—C13	56.6 (3)	C9—C8—C13—C12	0.4 (4)
C8—O2—C7—O1	6.1 (4)	O2—C8—C13—C12	175.6 (2)
C8—O2—C7—C4	−174.3 (2)	C13—C8—C9—C10	0.2 (4)
C6—C5—C4—C3	0.0 (4)	O2—C8—C9—C10	−175.2 (2)
C6—C5—C4—C7	−179.4 (2)	C8—C13—C12—C11	−0.6 (4)
C2—C3—C4—C5	0.3 (4)	C10—C11—C12—C13	0.2 (5)
C2—C3—C4—C7	179.7 (2)	C14—C11—C12—C13	179.8 (3)
O1—C7—C4—C5	−3.8 (4)	C8—C9—C10—C11	−0.6 (5)
O2—C7—C4—C5	176.6 (2)	C12—C11—C10—C9	0.4 (5)
O1—C7—C4—C3	176.7 (3)	C14—C11—C10—C9	−179.2 (3)

### Hydrogen-bond geometry (Å, °)

**Table:**

*D*—H···*A*	*D*—H	H···*A*	*D*···*A*	*D*—H···*A*
C3—H3···O1^i^	0.93	2.67	3.483 (4)	147.
C13—H13···O1^ii^	0.93	2.77	3.422 (4)	128.

Symmetry codes: (i) −*x*+2, *y*+1/2, −*z*+1/2; (ii) *x*, −*y*+3/2, *z*−1/2.
